# 4252 Automated Fetal Brain Volumetry on Clinical Fetal MRI Using Convolutional Neural Network

**DOI:** 10.1017/cts.2020.169

**Published:** 2020-07-29

**Authors:** Carol Tran, Orit Glenn, Christopher Hess, Andreas Rauschecker

**Affiliations:** 1 University Of California, San Francisco

## Abstract

OBJECTIVES/GOALS: We seek to develop an automated deep learning-based method for segmentation and volumetric quantification of the fetal brain on T2-weighted fetal MRIs. We will evaluate the performance of the algorithm by comparing it to gold standard manual segmentations. The method will be used to create a normative sample of brain volumes across gestational ages. METHODS/STUDY POPULATION: We will adapt a U-Net convolutional neural network architecture for fetal brain MRIs using 3D volumes. After re-sampling 2D fetal brain acquisitions to 3mm^3^ 3D volumes using linear interpolation, the network will be trained to perform automated brain segmentation on 40 randomly-sampled, normal fetal brain MRI scans of singleton pregnancies. Training will be performed in 3 acquisition planes (axial, coronal, sagittal). Performance will be evaluated on 10 test MRIs (in 3 acquisition planes, 30 total test samples) using Dice scores, compared to radiologists’ manual segmentations. The algorithm’s performance on measuring total brain volume will also be evaluated. RESULTS/ANTICIPATED RESULTS: Based on the success of prior U-net architectures for volumetric segmentation tasks in medical imaging (e.g. Duong et al., 2019), we anticipate that the convolutional neural network will accurately provide segmentations and associated volumetry of fetal brains in fractions of a second. We anticipate median Dice scores greater than 0.8 across our test sample. Once validated, the method will retrospectively generate a normative database of over 1500 fetal brain volumes across gestational ages (18 weeks to 30 weeks) collected at our institution. DISCUSSION/SIGNIFICANCE OF IMPACT: Quantitative estimates of brain volume, and deviations from normative data, would be a major advancement in objective clinical assessments of fetal MRI. Such data can currently only be obtained through laborious manual segmentations; automated deep learning methods have the potential to reduce the time and cost of this process.

